# Dyslipidemia and non-small cell lung cancer risk in Chinese population: a case-control study

**DOI:** 10.1186/s12944-018-0925-z

**Published:** 2018-12-06

**Authors:** Bo Hao, Miaomei Yu, Chen Sang, Baochen Bi, Jiajun Chen

**Affiliations:** 1 Department of Cardiothoracic Surgery, Xiangyang Central Hospital, Affiliated Hospital of Hubei University of Arts and Science, Xiangyang, 441021 China; 2 Comprehensive Laboratory, the Third Affiliated Hospital of Soochow University, Changzhou, 213003 China; 3 Department of Cardiothoracic Surgery, the Third Affiliated Hospital of Soochow University, Changzhou, 213003 China

**Keywords:** Dyslipidemia, Risk, Non-small cell lung cancer, Triglyceride, High-density lipoprotein cholesterol

## Abstract

**Background:**

Numerous studies reported that dyslipidemia was associated with cancer risk. However, few studies investigated the associations between dyslipidemia and non-small cell lung cancer (NSCLC).

**Methods:**

Four hundred twenty-four histologically confirmed NSCLC cases and 414 controls, matched for age and sex, were enrolled to examine the relationship between dyslipidemia and NSCLC. Demographic and clinical data were obtained from patients’ medical records and telephone interviews. Odds ratios (ORs) and 95% confidence intervals (CIs) were estimated using unconditional logistic regression.

**Results:**

Abnormal triglyceride (TG) and high-density lipoprotein cholesterol (HDL-C) levels showed statistically significant coexistence with NSCLC compared with controls. Higher levels of TG were associated with a higher risk of NSCLC (OR = 1.541, 95% CI, (1.072–2.215)). The odds ratios (ORs) for NSCLC for normal and high levels of HDL-C versus those with a low level of HDL-C were 0.337(95% CI, (0.242–0.468)) and 0.288(95% CI, (0.185–0.448)), respectively. After adjustment for age, sex, smoking status, hypertension, body mass index, diabetes and lipid profiles, the adjusted OR for normal and high levels of HDL-C were 0.320(95% CI, (0.218–0.470)) and 0.233(95% CI, (0.134–0.407)), respectively. However, after adjustment, high levels of TG increased the risk of NSCLC but not significantly (OR = 1.052, 95% CI (0.671–1.649)).

**Conclusions:**

This study provided evidence that dyslipidemia increased the risk of NSCLC in Chinese population.

## Background

Lung cancer has become the leading cause of cancer-related death in the worldwide, although progress in combating the disease has been made [1]. Non-small-cell lung cancer (NSCLC) is the most common subtype of lung cancer, which accounts for 80% of all cases [2]. It is estimated that 609,640 Americans will die of cancer in 2018, 25% of which are caused by lung cancer [1]. However, the exact mechanisms responsible for NSCLC remain unclear. In recent years, researchers held that the tumorigenesis in the body was a systemic disease [3]. Thus, more and more attention was paid to dysfunction and abnormality of metabolism caused by the etiology and pathogenesis of tumor [4]. And accumulating evidence suggested that altered lifestyle and obesity were closely linked to an increased risk of lung cancer [5, 6].

Cholesterol plays key roles in many vital physiological process which is involved in various biological functions, such as cell proliferation, division of both normal and tumor cells and the activity of membrane-bound enzymes [7]. Lipoprotein receptors, located on the cell surface, mediate cellular uptake and regulation of cholesterol. It has been noted that altered blood cholesterol levels were observed in some malignancies [8, 9]. Researchers also have found that abnormal levels of serum lipids and lipoproteins were associated with different types of cancers [10–12], despite the fact that increased lipids are closely related to the pathogenesis of coronary heart disease.

Numerous studies demonstrated that dyslipidemia was involved in carcinogenesis of various cancers. Chandler et al. [13] found that abnormal serum levels of lipids were associated with various cancer risks in women in a large cohort and a higher density lipoprotein cholesterol (HDL-C) level was linked to reduced cancer risk. A study by Agnoli et al. [14] indicated that high serum levels of low density lipoprotein cholesterol (LDL-C) and total cholesterol (TC) increased the risk of suffering colorectal cancer, particularly in men and postmenopausal women. Moreover, there is growing evidence showing that elevated serum levels of triglyceride (TG) and TC are associated with the increased risk of prostate cancer [15] and decreased HDL-C levels are associated with the reduced risk of non-Hodgkin lymphoma [16] and breast cancer [17]. However, so far, few studies investigated the roles of the lipid profiles play in NSCLC. And, thus, our study aims to explore the potential relations between dyslipidemia and NSCLC.

## Methods

### Patients

We retrospectively collected 424 newly diagnosed and pathologically confirmed NSCLC patients between 2016 and 2018 at the Department of Cardiothoracic at Xiangyang Center Hospital, the Affiliated Hospital of Hubei University of Arts and Science, and the Third Affiliated Hospital of Soochow University. And, 414 the population-based controls identified from age- and sex-matched were collected from Physical Examination Center, the Third Affiliated Hospital of Soochow University. The controls had no history of pneumonectomy or previous cancer. Of note, there were no statistically significant differences between the NSCLC group and the control group on age and sex when choosing matched control cases.

### Risk factor definition

The criterions for risk factor were as follow: 1) diabetes, fasting plasma glucose ≥6.1 mmol/L or 2-h plasma glucose ≥7.8 mmol/L, or a report of diabetes diagnosis from a physician; 2) hypertension, systolic blood pressure ≥ 140 mmHg or diastolic blood pressure ≥ 90 mmHg; 3) body mass index (BMI) ≥25 kg/m^2^ was considered as overweight; 4) alcohol drinking (yes) meant average daily alcohol consumption no less than 25 g alcohol for at least 1 year; 5) smoking status (yes) referred to current smokers or former smokers who had smoked no less than 100 cigarettes 1 year. 6) Statin use (yes) denoted patients who were using or received statin medications at least 30 days (simvastatin, pravastatin, atorvastatin, rosuvastatin, or fluvastatin).

Lipid profiles were stratified according to The Guidelines on Prevention and Treatment of Blood Lipid Abnormality in Chinese Adults (Version 2016).

### Data collection

Clinical data were obtained from the participants’ medical records and telephone interviews. The necessary clinical data of each enrolled participant were collected as follows: age at diagnosis, sex, weight, height, history of diabetes mellitus (DM), history of hypertension (HTN), the level of lipid profiles at diagnosis, smoking history, alcohol use and statins use. For patients with NSCLC, other clinical characteristics, such as tumor stage, pathological type and TNM stage, were also collected.

### Statistical analyses

Differences in categorical variables were analyzed by Chi-squared tests, and Student’s t-tests were used for continuous variables. P trends in lipid profile rankings were calculated by Kendall’s τ tests. Odds ratios (ORs) and its 95% CIs were calculated by unconditional multiple logistic regression, including terms for age, sex, BMI, history of smoking, history of hypertension, history of diabetes and statins use. The *P* values were two-sided and *P* < 0.05 was considered statistically significant in all statistical tests. SPSS 22.0 software (IBM Corporation, Armonk, NY, USA) was used for statistical analyses.

## Results

The characteristics of all participants were shown in the Table 1. The current study enrolled 424 patients newly diagnosed NSCLC patients and 414 controls. Among those participants, the mean (range) ages were 62.35 (32–79) years in NSCLC cases and the mean (range) ages were 61.57 (30–89) years in controls. 273 of NSCLC cases were males and 151 were female. In 414 controls, 280 were males and 134 were females.

**Table 1:** Demographic and clinical characteristics of 424 NSCLC cases and 414 controls

Variable	No. of Patients (%)			*P* value
	838	Controls (*n* = 414)	NSCLC (*n* = 424)	
Age (years)				0.886
≤ 45	36 (62.9)	19 (4.6)	17 (4.0)	
> 45& ≤ 65	451 (37.1)	224 (54.1)	227 (53.5)	
> 65	351 (37.1)	171 (41.3)	180 (42.5)	
Gender				0.321
Male	553 (62.2)	280 (67.6)	273 (64.4)	
Female	285 (37.8)	134 (32.4)	151 (35.6)	
BMI, Kg/m^2^				0.451
≤ 25	579 (69.1)	281 (67.9)	295 (70.3)	
> 25	259 (30.9)	133 (32.1)	126 (29.7)	
Smoking status				
No	663 (79.1)	383 (93.0)	280 (98.5)	< 0.001*
Ever/current	175 (20.9)	31 (7.0)	144 (1.5)	
Alcohol consumption				0.859
No	674 (80.4)	334 (11.8)	340 (23.1)	
Yes	164 (19.6)	80 (44.1)	84 (50.9)	
Hypertension				0.289
No	589 (70.3)	298 (72)	291 (68.6)	
Yes	249 (29.7)	40 (28)	108 (31.4)	
Diabetes				0.225
No	787 (93.9)	393 (78.9)	394 (92.7)	
Yes	51 (6.1)	21 (21.1)	30 (7.3)	
Statins use				0.583
No	813 (94.9)	403 (87.3)	410 (96.5)	
Yes	25 (5.1)	11 (12.7)	14 (3.5)	
Lipid profiles, mmol/L				
TC				0.295
Normal (< 5.2)	632 (75.5)	313 (75.6)	319 (75.4)	
BH (≥5.2& < 6.2)	159 (19)	83 (20.0)	76 (18.0)	
High (≥6.2)	46 (5.5)	18 (4.4)	28 (6.6)	
LDL-C				0.691
Normal (< 3.4)	772 (29.1)	382 (92.3)	390 (92.0)	
BH (≥3.4& < 4.1)	58 (55.0)	27 (6.5)	31 (7.3)	
High (≥4.1)	8 (15.9)	5 (1.2)	3 (0.7)	
HDL-C				< 0.001*
Low (< 1.0)	244 (29.1)	74 (17.9)	170 (40.1)	
Nomal (≥1.0& < 1.5)	461 (55.0)	260 (62.8)	201 (47.4)	
High (≥1.5)	133 (15.9)	80 (19.3)	53 (12.5)	
TG				0.026
Normal (< 1.7)	525 (62.6)	278 (67.1)	247 (58.3)	
BH (≥1.7& < 2.3)	159 (19.0)	71 (17.1)	88 (20.8)	
High (≥2.3)	154 (18.4)	65 (15.7)	89 (21.0)	
Pathological type				
squamous carcinoma	139 (32.8)			
adenocarcinoma	277 (65.3)			
others	8(1.9)			
TNM stage				
I	202 (47.6)			
II	85 (20.1)			
III	115 (27.1)			
IV	22 (5.2)			

^*^indicates that the difference was statistically significant

Abbreviation: *BH* borderline high, *BMI* body mass index, *TC* total cholesterol, *LDL-C* low density lipoprotein cholesterol, *HDL-C* high density lipoprotein cholesterol, *TG* triglyceride, *TNM* tumor-node-metastasis, *NSCLC* non-small cell lung cancer

We found no significant differences in age, sex, statin usage, smoking status, hypertension, history of diabetes or alcohol consumption between groups (Table 1). NSCLC patients had a significant prevalence of a habit of smoking (*P* < 0.001). Disorders of TG and HDL-C levels showed statistically significant coexistence with NSCLC.

Ever/current smokers showed a 5.5-fold increased possibility for suffering NSCLC compared with never smokers in univariate analysis (OR = 6.583, 95% CI (4.351–10.043)) (Tables 2 and 3). After adjusting for age, gender, BMI, alcohol consumption, history of hypertension, history of diabetes, statin use, HDL-C, TC, LDL-C, and TG, the multivariable OR = 15.979 (95% CI (8.880–28.752)). The results of univariate logistic regression analysis showed that a significant correlation between a high level of TG and NSCLC risk (OR = 1.541, 95% CI (1.072–2.215)). However, the multivariable analysis indicated that the association between a high level of TG and NSCLC was not significant (OR = 1.052, 95% CI (0.671–1.649)). Normal and higher levels of HDL-C (either univariate or multivariable logistic regression analyses) showed a significant association with a lower NSCLC risk (Normal: univariate OR = 0.337, 95% CI (0.242–0.468), multivariable OR = 0.320, 95% CI (0.218–0.470); High: univariate OR = 0.288, 95% CI (0.185–0.448), multivariable OR = 0.223, 95% CI (0.134–0.407)).

**Table 2:** Logistic regression analysis of lipid profiles and NSCLC risk

Variable	Univariate			Multivariable^a^		
	OR	95% CI	*P*	OR	95%CI	*P*
BMI						
≤ 25	1			1		
> 25	0.893	0.666–1.198	0.451	0.709	0.500–1.004	0.053
Smoking status						
No	1			1		
Ever/current	**6.583**	**4.315–10.043**	*P* < 0.001	**15.979**	**8.880–28.752**	*P* < 0.001
Alcohol consumption						
No	1			1		
Yes	1.031	0.733–1.451	0.859	0.988	0.686–1.332	0.786
Hypertension						
No	1			1		
Yes	1.174	0.873–1.580		1.077	0.753–1.541	0.684
Diabetes						
No	1			1		
Yes	1.425	0.802–2.532	0.227	1.175	0.553–2.494	0.675
Statins use						
No	1			1		
Yes	1.251	0.561–2.789		0.692	0.238–2.009	0.499
TC						
Normal (< 5.2)	1			1		
BH (≥5.2& < 6.2)	0.655	0.335–1.209	0.176	1.363	0.872–2.128	0.174
High (≥6.2)	0.589	0.302–1.149	0.120	1.488	0.878–2.521	0.115
LDL-C						
Normal (< 3.4)	1			1		
BH (≥3.4& < 4.1)	1.125	0.659–1.920		0.647	0.293–1.427	0.281
High (≥4.1)	0.588	0.139–2.476		0.198	0.029–1.364	0.100
HDL-C						
Low (< 1.0)	1			1		
Normal (≥1.0& < 1.5)	**0.337**	**0.242–0.468**	***P*** **< 0.001**	**0.320**	**0.218–0.470**	***P*** **< 0.001**
High (≥1.5)	**0.288**	**0.185–0.448**	***P*** **< 0.001**	**0.233**	**0.134–0.407**	***P*** **< 0.001**
TG						
Normal (< 1.7)	1			1		
BH (≥1.7& < 2.3)	1.395	0.977–1.993	0.067	1.352	0.898–2.036	0.149
High (≥2.3)	**1.541**	**1.072–2.215**	**0.019**	1.052	0.671–1.649	0.826

Abbreviations: *BH* borderline high, *BMI* body mass index, *TC* total cholesterol, *LDL-C* low density lipoprotein cholesterol, *HDL-C* high density lipoprotein cholesterol, *TG* triglyceride, *NSCLC* non-small cell lung cancer

^a^ Multivariable-adjusted model: The individual components of the metabolic syndrome have been mutually adjusted, age, sex, smoking status, hypertension, body mass index, and diabetes

**Table 3:** Logistic regression analysis of coexistence of lipid profiles and NSCLC TNM stage

Lipid profiles (mmol/l)	Control (*n* = 414)	TNM I			TNM II			TNM III-IV		
		Cases (*n* = 202)	Crude OR (95% CI)	Adjusted OR (95% CI)	Cases (*n* = 85)	Crude OR (95% CI)	Adjusted OR (95% CI)	Cases (*n* = 137)	Crude OR	Adjusted OR
**HDL-C**										
Low (< 1.0)	74	65	1	1	37	1	1	68	1	1
Normal (≥1.0& < 1.5)	260	110	**0.486(0.326–0.725)**	**0.473(0.292–0.768)**	40	**0.308 (0.184–0.516)**	**0.254(0.134–0.480)**	57	**0.239(0.154–0.369)**	**0.204(0.120–0.348)**
High (≥1.5)	80	27	**0.370 (0.213–0.644)**	**0.271 (0.132–0.555)**	8	**0.200 (0.087–0.457)**	**0.144(0.052–0.398)**	12	**0.163(0.082–0.326)**	**0.108(0.046–0.256)**

Abbreviation: *HDL-C* high density lipoprotein cholesterol, *TNM* tumor-node-metastasis, *NSCLC* non-small cell lung cancer

Adjusted model: adjustment for TC, TG, LDL-C, age, sex, smoking status, hypertension, body mass index, and diabetes

To investigate whether serum HDL-C levels were associated with different TNM classification of NSCLC, patients were stratified on the basis of their clinical stages. The univariate model showed that patients at TNM I stage with a high level of HDL-C had a reduced risk of NSCLC (OR = 0.370, 95% CI (0.213–0.644)) (Table 3), and normal levels of HDL-C decreased the risks for NSCLC (OR = 0.486, 95% CI (0.326–0.725)). In a multivariable model, patients at TNM I stage with both normal and high levels of HDL-C had a decreased risk of NSCLC (normal: adjusted OR = 0.473, 95% CI (0.292–0.768); high: adjusted OR = 0.271, 95% CI (0.132–0.555)). Moreover, patients with an elevated level of HDL-C had a decreased possibility for TNM II-IV NSCLC (TNM II: OR = 0.200, 95% CI (0.087–0.457), TNM III-IV: OR = 0.163, 95% CI (0.082–0.326)).

## Discussion

Dyslipidemia has been reported to be a risk factor for various cancers, such as breast, colorectal and prostate cancer [18–20]. In our current study, abnormal lipid profiles were found prevalent in patients with NSCLC. After adjusting for several lipid indicators and other potential risk factors for NSCLC, the coexistence persisted. It has been reported that a significant correlation between a lower HDL-C level and a higher risk of incident cancer, which was independent of patient age, sex, BMI, LDL-C levels, and the presence of diabetes and smoking [21]. Our prospective cohort study observed that normal and higher levels of HDL-C were associated with lower risks of NSCLC compared with a lower level (Normal: univariate OR = 0.337, 95% CI (0.242–0.468), multivariable OR = 0.320, 95% CI (0.218, 0.470); High: univariate OR = 0.288, 95% CI (0.185–0.448), multivariable OR = 0.223, 95% CI (0.134, 0.407)).

Cholesterol, one of the components of the membrane, plays a crucial role in maintaining the integrity of the cell membrane [22]. In addition, cholesterol was reported to accumulate in specific domains of the membrane and combine with sphingolipids, which formed compartmentalized and highly stable micro-domains termed lipid-rafts [23]. Epidermal Growth Factor Receptor (EGFR), one of growth factor signaling receptors, located in cholesterol-rich domains in the membrane, was associated with lipid-raft and involved in lung cancer [24]. HDL-C plays a key role in the process of reverse cholesterol transport, removes cholesterol from peripheral tissues and transports it to the liver for metabolism [25]. Wolfe et al. [26] held that cholesterol-enriched breast cancer cell membranes promoted EGFR signaling via lipid-raft formation and HDL-C suppressed the signaling mediated by lipid-raft via removing cholesterol from the membrane. Recently, a study by Su et al. [27] showed that HDL mimetics reduced viability and proliferation and decreased cell-mediated tumor burden in BALB/c mice. There was likewise a report showed that the same mimetic peptide yielded similar results in ovarian cancer [28]. In addition, emerging evidence suggested that low HDL-C levels were closely related to cancer incidence, including breast cancer [29], ovarian cancer [30], prostate cancer [31] and colon cancer [32].The reason for the decreased levels of HDL-C in NSCLC may be that an increased demand for additional cholesterol to support the growth of tumor tissues and membrane biosynthesis.

Several published studies, aiming to evaluate the relationship of TC and TG with NSCLC, drew inconsistent conclusions. In 2000, Siemianowicz et al. [33] clarified that TG concentration was lower in squamous cell lung cancer in a small cohort (the number of squamous cell lung cancer patients was 101). Their study also indicated that the serum levels of TC decreased in lung cancer patients, including NSCLC and small cell lung cancer. Chandler et al. [13] observed that high levels of TC and TG were associated with increased incidences of lung cancer in a woman’s cohort (the number of the cases was 190), while the association was not significant in a multivariable logistic regression model. We observed a modest association of high TG levels with increased incidences of NSCLC (univariate: OR = 1.541, 95% CI (1.072–2.215)) compared with control, while the association was not significant in a multivariable logistic regression analysis (multivariable OR = 1.052, 95% CI (0.671–1.649)). Compared with above-mentioned studies, we believe that our recruited participants might be more representative.

The present retrospective study only investigated the association between dyslipidemia and NSCLC and did not make causal inferences. Our current study would provide some implications for further research. The higher incidences of NSCLC occurred in developed Western countries. Meanwhile, lipid abnormalities were more prevalent in Western [34]. It is an interesting question that whether dyslipidemia is associated with the prevalence of NSCLC. Numerous studies suggested that lipid profiles could be altered by various factors which influenced the risk of cancer. Knight et al. [35] found that, although living in the same district, the lipid profiles of individuals were diverse in different ethnic groups. Moreover, a study showed that higher levels of TC were related to higher risks of prostate cancer and colon cancer in men and breast cancer in women, and increased levels of serum TC were related to decreased risks of liver cancer and gastric cancer in both men and women [36]. The findings above-mentioned and our study suggested that dyslipidemia may play different roles in NSCLC in different races, sexes and even tumor subtypes.

Our study observed a tumor-suppressing role of HDL-C and tumor-promoting role of TG in NSCLC. Thus, possible recommendations to encourage men to maintain healthy lipid profiles may reduce the NSCLC risk. Certainly, further researches from multicenter should be conducted to confirm our results in the future.

## Conclusion

Conclusively, abnormal HDL-C reduction and TG elevation were closely associated with a higher risk of NSCLC.
